# Removing Basis Set Incompleteness Error in Finite-Temperature Electronic Structure Calculations: Two-Electron Systems

**DOI:** 10.1021/acs.jpca.4c03769

**Published:** 2024-11-25

**Authors:** William Z. Van Benschoten, James J. Shepherd

**Affiliations:** Department of Chemistry, Michigan State University, East Lansing, Michigan 48824, United States

## Abstract

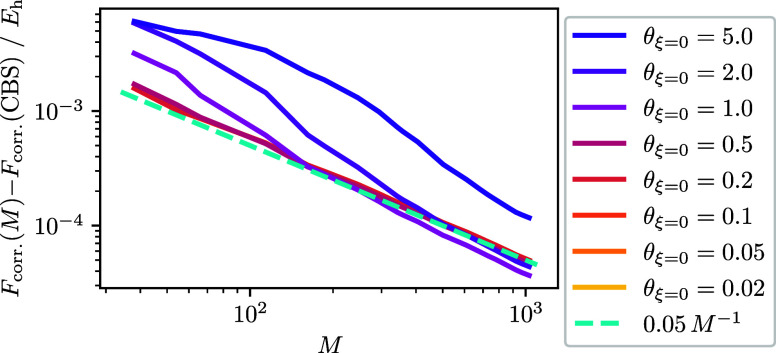

We investigate the basis-set-size dependence for quantities related to interacting electrons in the canonical ensemble. Calculations are performed using exact diagonalization (finite temperature full configuration interaction method) on two-electron model systems—the uniform electron gas (UEG) and the helium atom. Our data reproduce previous observations of a competition for how the internal energy converges between the ground-state correlation energy and the high-temperature kinetic energy. We explore how this can be related to component parts of the internal energy including kinetic, exchange, and correlation energies and show there is surprising nuance in how this can be broken down into mostly monotonically converging quantities. We also show that separation of the free energy into a free energy with/without correlation allows for monotonic convergence with basis set size due to the variational principle. We find that the free energy convergence matches the previously observed convergence properties of the internal energy. We discuss the free energy divergence that happens when converging a finite basis analytical hydrogen atom to the complete basis set limit and compare this to the energies of a helium atom in a large periodic box. Reducing the box size, we saw convergence trends for the helium atom that were similar to the UEG.

## 1 Introduction

While ground-state methods are very common in quantum chemistry software packages, finite temperature electronic structure is less ubiquitous and there is less consensus on how each method performs. There has been a rise in interest in the methods development community and there are methods which span a wide range of accuracy and cost.^1−10^ This is in part because of growing list of circumstances where finite electronic temperature is thought to be critical for describing nature accurately.^11−13^ Another factor is the added complexity for considering the entirety of the excited spectrum simultaneously. In the context of the growth of interest it is natural to devote some amount of inquiry into those things we take for granted in ground state methods within the context of finite electronic temperature.

One such consideration is truncated single-particle basis sets. In ground-state calculations, especially those that involve post-Hartree–Fock correlation, the basis sets developed by Dunning are ubiquitous^14^ as they resulted in a well-behaved procedure for improving and predicting energies at or near the ground state in the complete basis set (CBS) limit.^15^ In addition to this explicitly correlated methods (R12, F12, and transcorrelated methods) are becoming more common.^16−20^ One standard for finite-temperature electronic energies has been to converge temperature dependent quantities with basis set size to a relative error threshold^6,21−23^ but there are also papers that have used extrapolations based on various numerical analyses of the convergence of basis set incompleteness error (BSIE).^24,25^

Establishing BSIE for various levels of theory is complicated by factors such as the cost for increasing the basis set size as well as intricacies associated with the method. In the ground state, highly accurate methods such as full configuration interaction will be prohibited in the size of basis it can treat with modern computer resources as the memory cost grows exponentially. Basis set dependent quantum Monte Carlo (QMC) methods, such as auxiliary field QMC (AFQMC) and full configuration interaction QMC (FCIQMC), can reduce the cost to obtain highly accurate results. Removing basis set error in methods like AFQMC and FCIQMC can be complicated by stochastic error and approximations used therein, such as the phaseless and initiator approximations respectively.^26,27^ For perturbation theories there can be system dependent divergences.^28,29^ Lastly, for mean-field methods such as density functional theory (DFT) and Hartree–Fock theory (HF), basis set error is easier to overcome due to the low relative cost for the methods, but in some cases the methods are restrictive due to lower accuracy.^30^ Still there has been a great deal of work to treat BSIE at ground state (zero temperature limit) which has led to great insights resulting in cost saving measures to reduce BSIE.^15,31,32^ At finite temperature, things become complicated due to added expense and complicated behaviors resulting from temperature. Exact methods like finite temperature FCI are even more expensive, as the memory cost is squared relative to the ground state. This is also true for finite temperature QMC methods, and these can also take on additional severity and cost for moving to finite temperature.^33−36^ For perturbation theories, the divergence can persist, and there can be method inconsistencies.^37^ While mean-field methods are still considerably cheaper, one can still find the applications restrictive due to the lower accuracy.^38^ Thus, it is desirable to find cost saving measures for reducing BSIE at finite temperature similar to the ground state.

Two electron systems are important in the study of BSIE because the dominant source of error is the representation of cusps in the wave function using smooth functions,^39^ and these cusps occur when electrons are at short-range. In the study of ground state BSIE, two electron system have been analyzed in the demonstration of these cusp errors, the most famous of which is the helium atom,^40^ from which the 1/*X*^3^ relationship of the convergence of the Dunning basis sets can be found.^41^ In solids, the cusp conditions due to Kimball^42^ can be recast into a two-electron problem allowing for analysis on the electron gas demonstrating a similar power-law to molecules (1/*M* where *M* is the number of basis functions).^43^ This also led to the explicit parametrization of wave functions by functions involving the interelectron coordinate (R12 or F12 methods)^44^ which has also seen application to solids.^45^ In general, two electron systems can be powerful models whether they are model Hamiltonians^46−50^ or small *ab initio* Hamiltonians.^31,51−56^

We are interested in studying the CBS convergence of several temperature dependent quantities using two electron systems because, for this few electrons, it is possible to perform FCI/exact diagonalization on a wide range of basis set sizes. Temperature dependent quantities in a canonical ensemble are calculated exactly using the finite temperature full configuration interaction method (ft-FCI).^57^ The quantities investigated include the typical thermodynamic quantities for the canonical ensemble, which are the: internal energy, entropy, and free energy. In addition to this, we separate the internal energy into the kinetic and potential energy components, as well the potential energy into the exchange and correlation components. We also define a free energy of correlation by analogy to the ground-state correlation energy and show how it can be useful for examining basis set convergence. Finally, we also investigate the convergence behavior for the momentum distribution and static structure factor (SSF).

Our main choice of Hamiltonian is the uniform electron gas (UEG) described by a plane-wave basis corresponding to a simple-cubic lattice symmetry.^58^ We also consider the helium atom under periodic boundary conditions (PBC) at two different box sizes. Our goal when examining atoms is to highlight any differences between what we find and the divergence in the electronic free energy of a hydrogen atom in a vacuum.^59−61^

## 2 Theory

### 2.1 Thermodynamic Quantities

In this section we adopt formula and some notation conventions from ref (57). Throughout this section and the remainder of this paper, we work with Hartree atomic units unless otherwise stated.

For a canonical ensemble of *N* electrons, we write the electronic Hamiltonian using a finite Slater determinant basis as
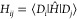
1which we exactly diagonalize to produce the orthogonal wave function solutions
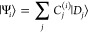
2also known as the full configuration interaction solutions. These satisfy the Schrödinger equation
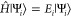
3

Given the wave function solutions we write the *N*-electron density matrix as a sum over states
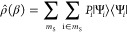
4where we use *m*_S_ to represent all the possible spin magnetic quantum numbers within the *N*-electron determinant basis and *P*_*i*_ is the thermal weight for state *i*. We calculate the quantity *m*_S_ as
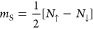
5where *N*_↑_ is the number of α electrons and *N*_↓_ is the number of β electrons in the Slater determinant. Then the valid quantities for *m*_S_ in eq 4 are found by ensuring that *N* = *N*_↑_ + *N*_↓_. Finally, the thermal weights are calculated as
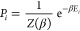
6where  is the thermodynamic temperature and *Z*(β) is the partition function
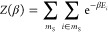
7

Given the density matrix, the expectation value for any operator at finite temperature is found as
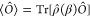
8such as the internal energy and the entropy
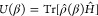
9
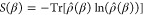
10Here we assume the normalized density matrix is used, and so exclude the  term in the denominator. As the density matrix in the FCI basis is diagonal, the internal energy and entropy can also be calculated as a sum over these states
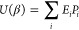
11
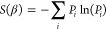
12Finally, the free energy can be calculated as
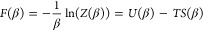
13

### 2.2 Uniform Electron Gas

The UEG, also known as jellium, is a model system which describes electrons under PBC subjected to a uniform positive background charge. The UEG is one of the simplest model systems for fermions as it can be parametrized by the electron number *N* and density parameter *r*_s_. The electron number and density parameter are related by the expression 4π*Nr*_s_^3^ = 3Ω, where Ω is the volume which is related to the length *L* = Ω^1/3^ for the periodic box.

In the UEG, the Hamiltonian operator can be written as
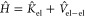
14where *K̂* and *V̂* are the kinetic and Coulomb interactions for/between electrons (el). Henceforth we forego including the el and el–el subscript.

We work with a single-particle basis of plane-waves in momentum space (*k*-space)
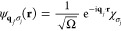
15where , σ_*j*_ is the spin (↑ or ↓) for the electron, **q**_*j*_ is a vector of integers scaled by 2π/*L*, and χ_σ_*j*__ is a unit vector for the spin. The grid is centered on the middle of *k*-space i.e. no twist angle offset is used. We note that our choice for the **q**_*j*_ coordinates corresponds to a simple-cubic symmetry, but one may also choose alternative coordinates such as body-centered or face-centered symmetries. These orbitals have a kinetic energy  and by limiting those orbitals in our plane-wave basis set to those equal to or below an energy cutoff (ϵ_cut_) we can define a finite basis.

The kinetic energy of a determinant is
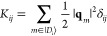
16where *m* is an occupied orbital in determinant |*D*_*i*_⟩ and δ_*ij*_ is the Kronecker delta function. This allows for the kinetic energy to be calculated using eq 8.

The potential operator has the normal Fourier components of
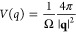
17and, for this work, the Madelung component (*q* = 0) is omitted.

Following notation from Dornheim et al., we define the reduced temperature as^62^
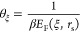
18where ξ = (*N*_↑_ – *N*_↓_)/*N* is the spin polarization parameter and *E*_F_ is the Fermi energy. Here we only use reduced temperatures corresponding to ξ = 0, which has no impact on the *m*_S_ used for the calculation. As such, the Fermi energy for eq 18 is calculated as
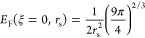
19

Appendix A describes the equations used to calculate the momentum distribution and SSF.

### 2.3 Note on Spin Polarization

When summing over states above, consideration needs to be given to spin polarization which can vary (*m*_S_ = −1, 0, 1). Here, |*m*_S_| = 1 implies two unpaired electrons. The canonical ensemble as commonly defined fixes particle number and this is normally interpreted as number of electrons (*N*) in finite-temperature electronic structure.^3,5,25,33,57,63−68^ Such a definition does not always fix the number of electrons of a given spin, leaving spin polarization to vary.^3,25,57,66^ It is possible to find both examples in the literature with respect to the canonical ensemble^3,5,25,33,57,65,66,68^ and it particularly important to consider this in the case of magnetic model Hamiltonians where it tends to be mentioned more explicitly.^65^ There is a more general discussion in ref (64) that suggests it is prudent to fix as many symmetries as possible in the canonical ensemble for practical reasons, provided there is some reason that it would agree with other ensembles in some limit.

Ordinarily, when studying BSIE it would be more natural to study the spin polarizations separately. However, when the spins are paired in the two-electron UEG there is not an exchange contribution which means that the finite-temperature exchange convergence would not be visible. This affects some of our analysis in Sections 4.2 and 4.5 below. In this way including both spin polarizations leads to a more interesting and rich narrative. Multielectron systems will include contributions from singlet and triplet cusps, so a discussion of what happens when they are both present is relevant (even if its manifestation will be different in multielectron systems). It is also the case that studies close to this one in our community, hence the target audience for our work, generally consider summing over spin polarizations^3,25,57,66^ if this is mentioned at all. Some of our prior work also sampled all spin polarizations.^36^

Ultimately, we chose to include all spin polarizations. The physical picture associated with this choice is that we are cooling the system down from a high temperature limit where all *m*_S_ sectors were available. If we think of the boundary of the system being simulated and its surroundings as enforcing the fixing of *N*, *V*, and *T*, then this is equivalent to allowing spin polarization to be affected at the boundary.

At *T* → 0, the system resembles the *m*_S_ = 0 ground state. As the HF kinetic and exchange energies are zero, the total kinetic and exchange energies are quite small. As temperature rises the system rapidly acquires |*m*_S_| = 1 character (i.e., two unpaired electrons) and this is why we continue to discuss exchange and spin-parallel correlation in our results below.

### 2.4 Prior Work on CBS Convergence at Finite Temperature

Prior work to estimate the CBS limit by extrapolation has identified that the convergence power law for the finite temperature internal energy has competing terms corresponding to a ground-state-like correlation energy term and a high-temperature kinetic energy term.^24,25^ Power laws for these components in the electron gas have been derived. For an unpolarized gas, the correlation energy converges to the CBS limit as *M*^–1^ (ref (43)), where *M* is the number of single-particle spin–orbitals. For a polarized gas, the correlation energy converges to the CBS limit as *M*^–5/3^ (refs (33 and 34)). In many-electron systems, the second power law here due to the spin polarized gas can also come from two-electron exchange-like contributions in the correlation energy. The noninteracting thermal kinetic energy, for sufficiently high temperatures, can be found to converge as .^24^ Here, and throughout, the Δ refers to energy differences. Thus Δ*K*^(0)^ is a noninteracting kinetic energy and refers to the missing energy for a given *M* relative to the CBS. We anticipate these results readily apply here for our UEG results, as we construct our Hamiltonian similarly with the same single-particle basis scheme.

For the BSIE in the correlation, both of these expressions can be derived by considering the leading order error term using methods like second-order Møller–Plesset perturbation theory (MP2)^43^ or the random phase approximation.^34^ As stated previously, the basis set is defined by ϵ_cut_, which defines a sphere where orbitals for **q** reside in the basis if |**q**|^2^/2 is within ϵ_cut_. In this way, the sphere has a radius like ϵ_cut_ = |**q**_cut_|^2^/2 ∝ *M*^2/3^. For the unpolarized system, the basis set incompleteness comes from a multiplicative factor between the energy for higher order excitations ∝1/|**q**|^4^ and the corresponding orbital energies ∝1/|**q**|^2^ for **q** > **q**_cut_ which are missed when restricting the basis set using ϵ_cut_. Using spherical integration over the missing **q** (**q**_cut_ < **q** < ∞), one finds the final error term is ∝1/|**q**_cut_|^3^ and substituting in |**q**_cut_|^2^ ∝ *M*^2/3^ we find *M*^–1^. In the case of polarized system, the only change is that the excitation term is instead ∝1/|**q**_cut_|^6^. Integration results in the error term ∝1/|**q**_cut_|^5^, and after substituting this gives *M*^–5/3^.

### 2.5 Computational Cost and CBS Convergence

For this work, we have used the exact FCI method, and store the entire determinant basis, Hamiltonian, and wave functions on disk in order to calculate the density matrix and other thermodynamic quantities on-the-fly. This requires tens of gigabytes of disk each for the larger basis set sizes. Additionally, our largest calculation took 500 gigabytes of memory and a 34 h to complete. We found that doubling the basis set size increased the compute time by a factor of roughly 10–20 times and memory requirement by a factor of 20–30 times. For evaluating thermodynamic quantities on-the-fly we found that symmetry could be used to reduce the amount of memory required to below 18 gigabytes and compute time to less than an hour per temperature. The memory and time requirements, and inherent scaling of each, highlights the importance for reducing the impact of basis set error without the need for performing a significant number of large *M* calculations.

## 3 Calculation Details

Exact diagonalization (FCI) calculations were carried out using HANDE-QMC and NumPy.^69,70^ The integrals and Hamiltonian files needed for the UEG are generated using HANDE-QMC. Calculations for thermodynamic quantities are performed with in house python scripts using NumPy and mpmath library routines.^70,71^ For generating fits for quantities to assumed functional equations we used the curve_fit routine within the SciPy library.^72^

All calculations use a Hilbert space containing all possible Slater determinants defined by exciting the *N* electrons for the system in *M* single-particle spin–orbitals. Unless stated otherwise, we include all possible spin polarizations in the Slater basis that conserve particle number.

Calculations for the UEG are done using the density parameter *r*_s_ = 1 or *r*_s_ = 10. For simulating the helium system using PBC we used the Vienna *ab initio* simulation package (VASP) to generate the integrals.^73^ Integrals are generated from a restricted Hartree–Fock calculation that uses a DFT calculation as the initial one-electron density guess. For all VASP calculations we used Fermi smearing with a smearing width of σ = 0.0001. We used the all electron POTAR file for helium: He_AE, as such no pseudopotential was used for our periodic calculations. All calculations with PBC are performed at the Γ-point for the Brillouin zone.

## 4 Results

### 4.1 Our Model System Reproduces a Turnover in the Internal Energy Convergence

We begin our investigation by examining the internal energy across a range of basis set sizes and temperatures. We start from the internal energy because it is most directly accessible from the Hamiltonian. We will consider a range of temperatures from θ_ξ=0_ = 0.02 to θ_ξ=0_ = 5.0 as to our observations these represent the different types of behavior we are interested in that can be encountered in the internal energy.

**Figure 1:**
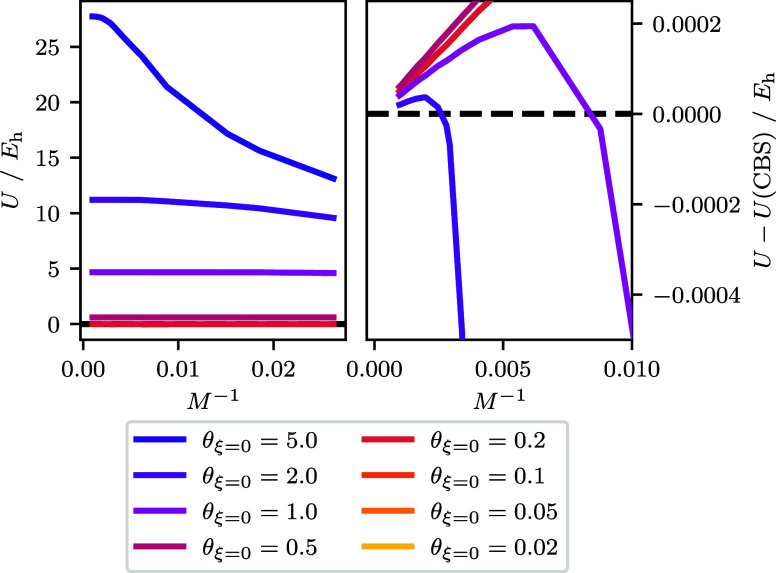
Internal energy as a function of spin–orbitals for the *r*_s_ = 1 UEG. Temperatures are calculated using eq 18 with ξ = 0 and *r*_s_ = 1. For the CBS extrapolation the largest three values of *M* are used, which corresponds to *M* ∈ {922, 970, 1030}. The largest basis size used *M* = 1030 corresponds to the left most point in either panel, while the smallest basis size *M* = 38 corresponds to the right most point and is only visible in the left panel. The right panel shows the total energies from the left panel with the extrapolated *U*(CBS) estimate subtracted. To facilitate discussion the right panel is shown using a smaller range for the axes which are based on the origin and for which the θ_ξ=0_ = 5.0 data fall outside and so are not visible. For θ_ξ=0_ ≤ 0.2 the data are similar enough to overlap resulting in only the θ_ξ=0_ = 0.2 curve being observed. For θ_ξ=0_ ≤ 2.0 the extrapolated *U*(CBS) error is ≤2 × 10^–6^*E*_h_, with smaller θ_ξ=0_ generally having smaller error, while for θ_ξ=0_ = 5.0 the error is ∼2 × 10^–3^*E*_h_.

Figure 1 shows the internal energy dependence on the inverse number of spin orbitals *M* for the two-electron UEG. At low temperatures we observe the internal energy is relatively constant across *M* for the energy scale being used. As the temperature increases from low to intermediate θ_ξ=0_, there is generally a constant upward shift in the internal energy across all *M*. This is because for these temperatures the internal energy is numerically dominated by the energy from small basis set sizes.

As the temperature rises to θ_ξ=0_ = 2 and θ_ξ=0_ = 5, the energy appears to converge to a larger value than found at small *M*. This is in contrast to the ground-state convergence where the variational principle forces a convergence from above with increasing *M*. At a certain *M* the curve appears to “shoulder”. At this point, the number of states that are thermally accessible is tapering off. The broader trend is that higher temperatures require more *M* before the tapering off happens, which is consistent with more states being available at higher temperatures.

In the right panel for Figure 1, we focus our attention on the behavior of *U* at high *M* by subtracting an estimate for the CBS limit *U*(*M* = ∞) and zooming in on the origin. To estimate the CBS we used the intercept resulting from a least squared regression applied to the energy equation *U*(*M*) = *aM*^–1^ + *b* using the three largest values of *M*. At θ_ξ=0_ < 1 the internal energy approaches the CBS from above linearly with *M*^–1^ across all *M*. This result is reasonable because for the smallest nonzero temperatures the system will still be dominated by the ground state.

For θ_ξ=0_ = 1.0 and θ_ξ=0_ = 2.0 the difference is observed to cross between negative and positive. When the difference is negative the internal energy is approaching the CBS estimate from below, and when the difference is positive the internal energy is approaching the CBS from above. This result implies that high *M* will tend back toward the ground-state CBS limit power-law, even though the energy might be imperceptibly small at high temperatures. This has been observed by Malone, where they determined that the ground-state behavior returns near ,^34^ which is consistent with our findings. At θ_ξ=0_ = 5 the internal energy is still increasing with decreasing *M*^–1^ even for the largest basis set size. Though the turnover appears to nearly have emerged in the left panel of Figure 1, extrapolating from too small of a basis set using *M*^–1^ could result in an extrapolated energy that is low or high depending on d*U*/d*M*^–1^.

### 4.2 Components of the Internal Energy

In order to analyze the properties of the internal energy convergence, we separate the internal energy into the following components
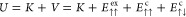
20and also use the following relationship
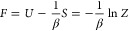
21Here *E*_↑↑_^ex^, *E*_↑↑_^c^, and *E*_↑↓_^c^ are the parallel spin exchange energy, parallel spin correlation energy, and antiparallel spin correlation energy respectively (defined in Appendix A).

**Figure 2:**
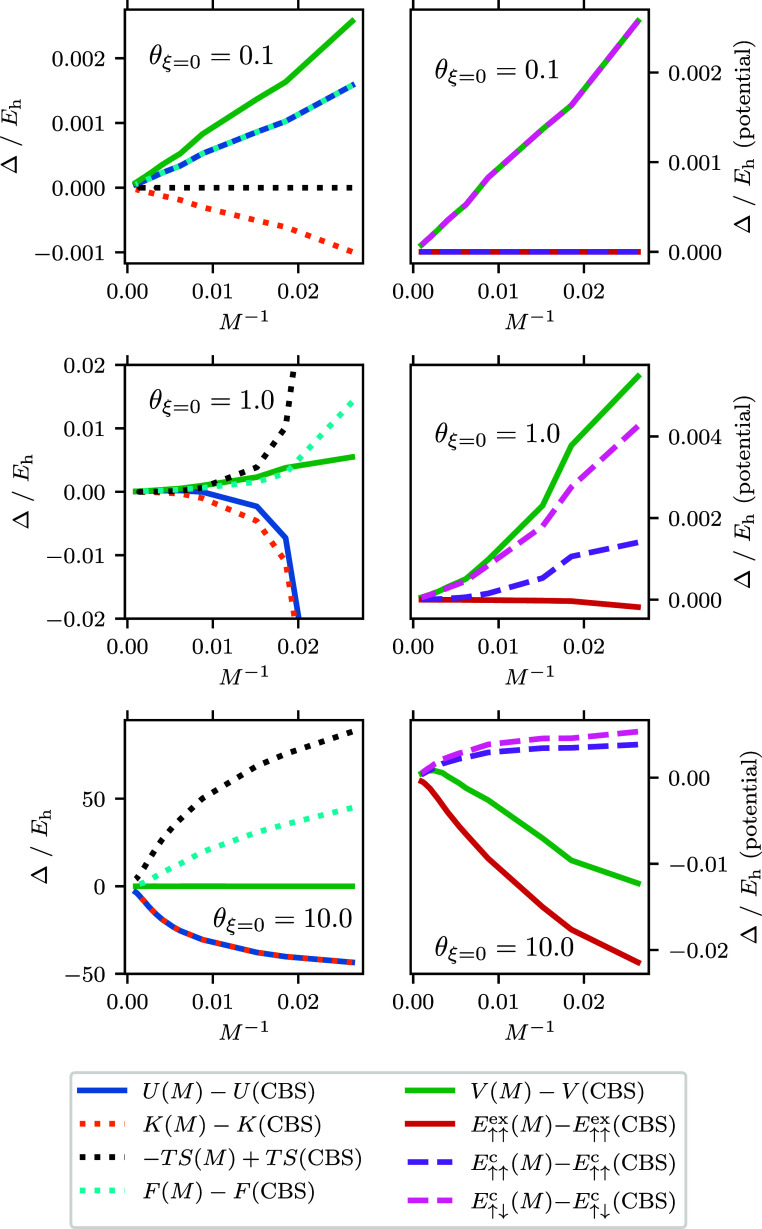
Various energy differences are plotted against *M*^–1^ for the *r*_s_ = 1 UEG. Each energy is a component of the free energy (for details, see text). Here Δ refers to the difference between the energy at finite *M* and the CBS limit is referred to as the BSIE. For each quantity shown, the CBS estimate used for the difference was calculated using an extrapolation performed on that quantity assuming a *M*^–1^ convergence. The right panels show the components of the potential as these are on a different energy scale for this system. For the CBS extrapolation the largest three values of *M* are used, which corresponds to *M* ∈ {922, 970, 1030}. The largest basis size used *M* = 1030 corresponds to the left most point, while the smallest basis size *M* = 38 corresponds to the right most point, for all panels. For θ_ξ=0_ = 0.1 and θ_ξ=0_ = 1.0 the CBS extrapolations have errors ≤3 × 10^–6^*E*_h_ for all energies. For θ_ξ=0_ = 10.0, the CBS extrapolation error for energies in the left panel are ∼1 × 10^–1^*E*_h_ except for *F*(CBS) which is ∼3 × 10^–2^*E*_h_, and similarly for the right panel the errors are roughly in the range (2–3) × 10^–5^*E*_h_.

Figure 2 shows examples of the CBS limit convergence for three different representative temperatures: θ_ξ=0_ = 0.1, 1.0, and 10.0. These are low, intermediate, and high temperatures respectively. Several quantities appear to converge monotonically and from the same direction in these graphs: *K* and *E*_↑↑_^ex^ converge from below; *F*, *E*_↑↑_^c^, and *E*_↑↓_^c^ converge from above. The other quantities, −*TS*, *U*, and *V*, all can be seen to converge nonmonotonically or change the direction of their convergence between different temperatures.

At θ_ξ=0_ = 0.1: the internal energy convergence is from above and behaves as *M*^–1^. There is a significant amount of BSIE, which we consider to be the difference between the CBS estimate from extrapolation and a finite *M* estimate. The BSIE comes from *K* as well as *V* but for most of the range of basis set sizes this is a linear trend in *M*^–1^. The linear component in *V* comes from the ground-state-like convergence of the correlation energy, specifically *E*_↑↓_^c^. At low temperatures the contribution from the |*m*_S_| = 1 is small and the *E*_↑↑_^c^ and *E*_↑↑_^ex^ components do not have appreciable BSIE.

At θ_ξ=0_ = 1.0: the internal energy convergence is mostly from below though close to the origin there is a turn-over (visible in the right panel of Figure 1). Here, the internal energy is mostly the kinetic energy component. The turnover comes from *E*_↑↑_^c^ and *E*_↑↓_^c^ which are of a similar order of magnitude to *E*_↑↓_^c^ at θ_ξ=0_ = 0.1 and only affect the convergence of *U* in a minor way for this system. That said, *E*_↑↑_^c^ and *E*_↑↓_^c^ do cause *V* to converge from above. The shape of the curve for *U* matches the high *M*^–1^ behavior of , the noninteracting kinetic energy BSIE, which is rapidly convergent in *M*.

At θ_ξ=0_ = 10.0: the internal energy convergence is now from below and follows the trend for the kinetic energy. The exchange contribution *E*_↑↑_^ex^ now competes with the correlation contributions and cause *V* to converge from below, with a small change in direction close to the CBS limit. The shape of the curve for *U* matches the intermediate *M* behavior of , and there is a risk for a *M*^–1^ extrapolation to significantly underestimate the CBS limit. This indicates that the assumed *M*^–1^ convergence is no longer the appropriate convergence rate.

**Figure 3:**
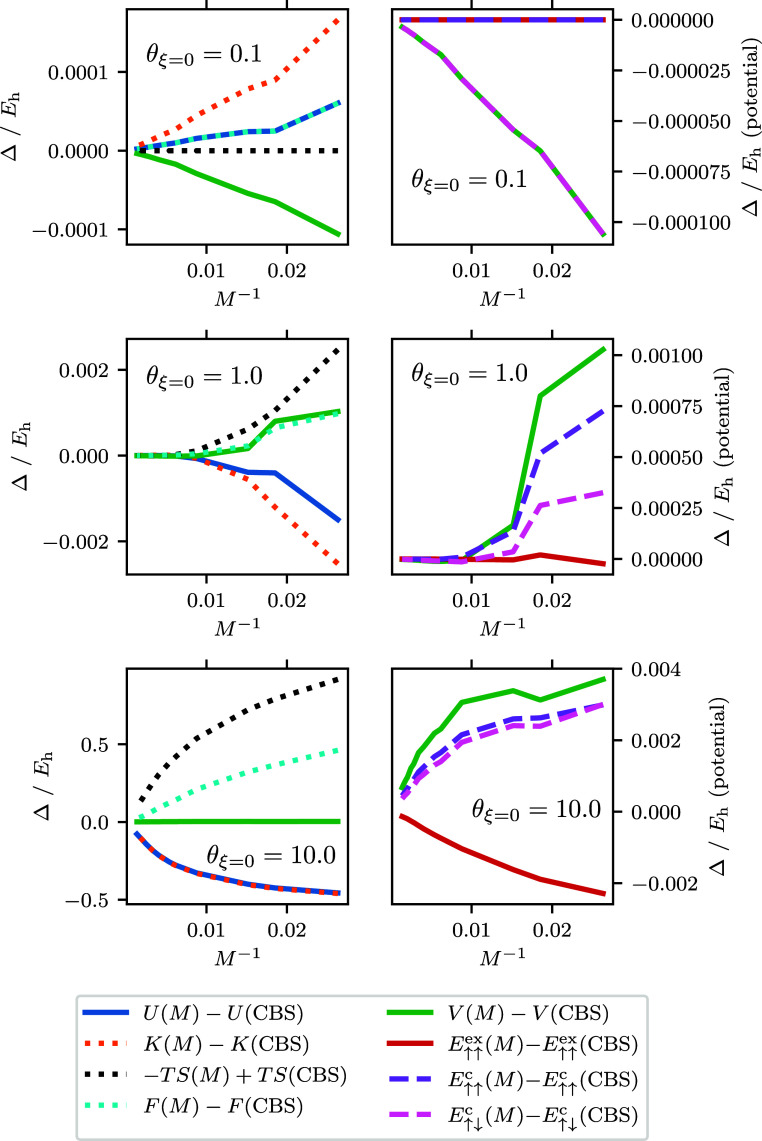
Various energy differences are plotted against *M*^–1^ for the *r*_s_ = 10 UEG. Each energy is a component of the free energy (for details, see text). Here Δ refers to the difference between the energy at finite *M* and the CBS limit is referred to as the BSIE. For each quantity shown, the CBS estimate used for the difference was calculated using an extrapolation performed on that quantity assuming a *M*^–1^ convergence. The right panels show the components of the potential as these are on a different energy scale for this system. For the CBS extrapolation the largest three values of *M* are used, which corresponds to *M* ∈ {610, 682, 730}. The largest basis size used *M* = 730 corresponds to the left most point, while the smallest basis size *M* = 38 corresponds to the right most point, for all panels. For θ_ξ=0_ = 0.1 and θ_ξ=0_ = 1.0 the CBS extrapolations have errors ≤6 × 10^–8^*E*_h_ for all energies. For θ_ξ=0_ = 10.0, the CBS extrapolation error for energies in the left panel range from (3–9) × 10^–4^*E*_h_, while in the right panel range from (3–5) × 10^–6^*E*_h_ except for *V*(CBS) which was ∼1 × 10^–5^*E*_h_.

We also compare *r*_s_ = 1.0 (Figure 2) with *r*_s_ = 10.0 (Figure 3): while the higher temperatures generally behave the same, θ_ξ=0_ = 0.1 is remarkably different. Here, the kinetic energy converges from above. We attribute this to correlation effects in the kinetic energy that are part of why the ground-state correlation converges from above (since the HF does not change with *M*). These contributions from relaxation could come from either the ground or excited states. The potential changes direction for a similar reason to the kinetic energy.

These graphs also highlight the free energy, *F*, converging from above. This is consistent with the variational principle in the canonical ensemble, *F*_trial_ ≥ *F*_exact_, where *F*_trial_ is the free energy of trial density matrix. We would expect this to hold upon adding basis functions to the system, provided the other conditions of the ensemble were met. This is the finite-temperature version of the ground state *E*_trial_ ≥ *E*_exact_. At θ_ξ=0_ = 0.1, the free energy is approximately the same as the internal energy. At θ_ξ=0_ = 1.0 and 10.0, the free energy behaves like the internal energy except that it converges from above. This is because the entropic contribution to the energy, −*TS*, converges from above and is a larger contributor to BSIE than the internal energy.

In summary, we see that there may be a difficulty in straightforward extrapolations to the CBS limit for intermediate-temperature-regime systems because there is a competition between kinetic and correlation contributions to the energy. Two different options emerged in our analysis. In the first, we found components of the internal energy can sometimes behave monotonically depending on simulation parameters (*r*_s_, θ_ξ=0_), suggesting that they can sometimes each be identified and extrapolated separately. The main component here would be to separate out the kinetic energy. The main drawback to this is that we saw when BSIE becomes small in magnitude there can be extremely complicated effects where no single component for the internal energy controls the convergence behavior and the behavior for the components are nonmonotonic. In the second, we saw that the free energy would converge monotonically to the CBS limit as a consequence of the variational principle, with the entropic component washing out this behavior in the internal energy.

### 4.3 Definition of the Free Energy of Correlation, and Determining How It Converges to the Complete Basis Set Limit

We saw in the previous section that the free energy is a useful quantity for extrapolation as it converges monotonically from above. Continuing this exploration, we can note that the Hamiltonian in a Slater determinant basis can be partitioned into a diagonal part, *H*^(0)^, and its off-diagonal part, *H*_I_
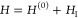
22When the diagonal part is used to form the density matrix (ρ^(0)^) this is a thermal occupation of the Slater determinants that come from a HF calculation. In the grand canonical ensemble this is significant because a simple reduced form for the thermal occupations of the orbitals exists (through Fermi–Dirac statistics).^3^ However, in the canonical ensemble, the equivalent result is much more complicated and, in our calculations, we use a Monte Carlo sampling of ρ^(0)^.^24^

The internal energy for the *H*^(0)^ density matrix can be above or below the true internal energy at finite temperature. Switching to the free energy allows us to take advantage of variationality^74^
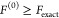
23to define an energy that is always negative
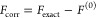
24In *F*^(0)^, the interaction is limited the mean-field interaction in Hartree–Fock theory and therefore *F*_corr_ is the free energy due to correlation. Specifically, we calculate *F*^(0)^ from eq 13 using *Z*^(0)^ as our partition function which we calculate with eq 7 using *H*_*ii*_ from eq 1 as the *E*_*i*_.

**Figure 4:**
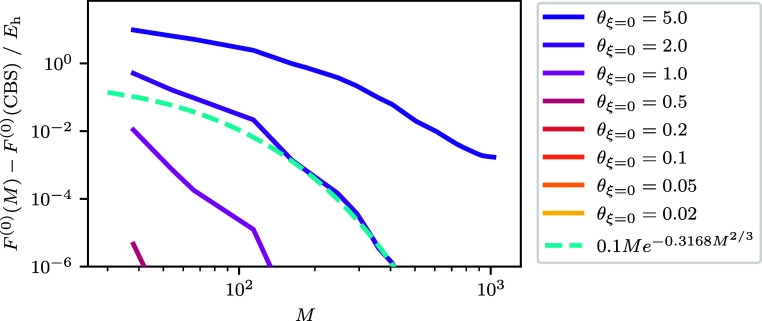
Noninteracting free energy difference to the best CBS estimate for the free energy as a function of spin–orbitals for the *r*_s_ = 1 UEG. The graph shows a dashed line of the form , the noninteracting kinetic energy BSIE. *C* is determined by fitting the expression ϵ_cut_ = *CM*^2/3^ + *b* using the pairs of ϵ_cut_ and *M* for the *r*_s_ = 1 UEG. The smallest and largest *M* for the data are *M* = 38 and *M* = 1030 respectively. For the CBS estimate *F*^(0)^(CBS) an extrapolation is performed using the largest three values of *M* which corresponds to *M* ∈ {922, 970, 1030}. For all but θ_ξ=0_ = 5.0, the *F*^(0)^(*M* = 1030) and *F*^(0)^(*M* = 970) energies are the same within our numerical precision, and so the error for the extrapolated CBS is not interpreted. For θ_ξ=0_ = 5.0 this has the effect that the *F*^(0)^(CBS) is to low, and so the difference appears to converge toward a nonzero value. The error for the θ_ξ=0_ = 5.0 extrapolated CBS estimate is ∼2 × 10^–4^*E*_h_. For θ_ξ=0_ ≤ 0.2 the data fall outside the range shown and are not visible.

**Figure 5:**
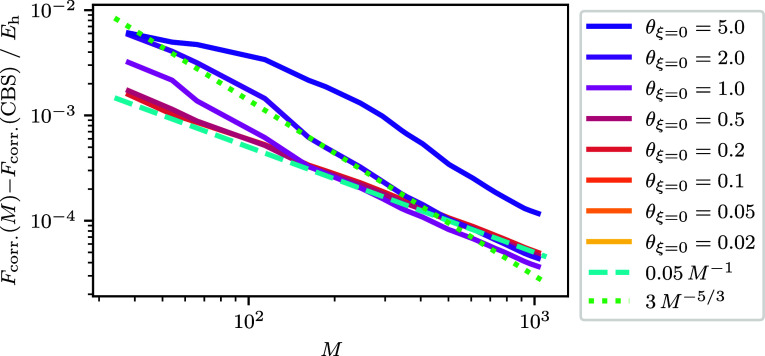
Free correlation energy difference to the best CBS estimate for the free correlation energy as a function of spin–orbitals for the *r*_s_ = 1 UEG. The smallest and largest *M* for the data are *M* = 38 and *M* = 1030 respectively. For the CBS estimate *F*_corr_(CBS) an extrapolation is performed using the largest three values of *M* which corresponds to *M* ∈ {922, 970, 1030}. The error for the extrapolated CBS estimates generally increase across θ_ξ=0_ and range from ∼2 × 10^–7^*E*_h_ for the smallest θ_ξ=0_ to ∼3 × 10^–6^*E*_h_ for θ_ξ=0_ = 5.0.

The quantities *F*^(0)^ and *F*_corr_ both would be expected to converge to the CBS limit from above. When more basis functions are added the lack of orbital relaxation in the UEG means that this effectively adds more terms to *H*_*ij*_ and ρ_*ij*_. In the case of *F*^(0)^, *Z*^(0)^ is being increased for each basis function added because more terms are added from *H*_*ii*_ without any being removed. In the case of *F*_corr_ the monotonicity seems to depend on how the terms affect the FCI energies that have already been added. Strict monotonicity on changing from a smaller to a larger basis set would require that ρ_*ij*_ is solved for in a way that chooses the ρ_*ij*_ with the lowest energy from an available set of solutions and that it is possible for the solver to find the solution for the smaller basis set size with additional zeros for the new states (thus having the same free energy). Figures 4 and 5 show how these two quantities converge to the CBS limit. Once again it is possible to see the three regimes. At low temperatures, *F*_corr_ determines the basis set convergence, which follows a similar trend to the ground-state correlation energy. This is so much the case that for Θ < 0.2 the lines showing the BSIE are overlapped. At high temperatures, *F*^(0)^ contributes most to the energy and basis set convergence. Here, convergence is exponentially fast similar to the high temperature convergence of the internal energy. This can be verified by comparing the convergence to a line with the formula , the same as we used for the noninteracting kinetic energy. When there are enough basis functions the convergence will return to being driven by *F*_corr_, but the energy contribution of this will be low in comparison to *F*^(0)^. At intermediate temperatures, it is conceivable there could be a combination of both behaviors seen in the energy as the convergence of *F*_corr_ and *F*^(0)^ are more comparable. At θ_ξ=0_ = 2.0 we can see most clearly a turnover just before *M*^–1^ which corresponds to *M*^–5/3^, which corresponds to |*m*_S_| = 1 contributions.

### 4.4 Relationship between Systems under PBC and a Vacuum

**Figure 6:**
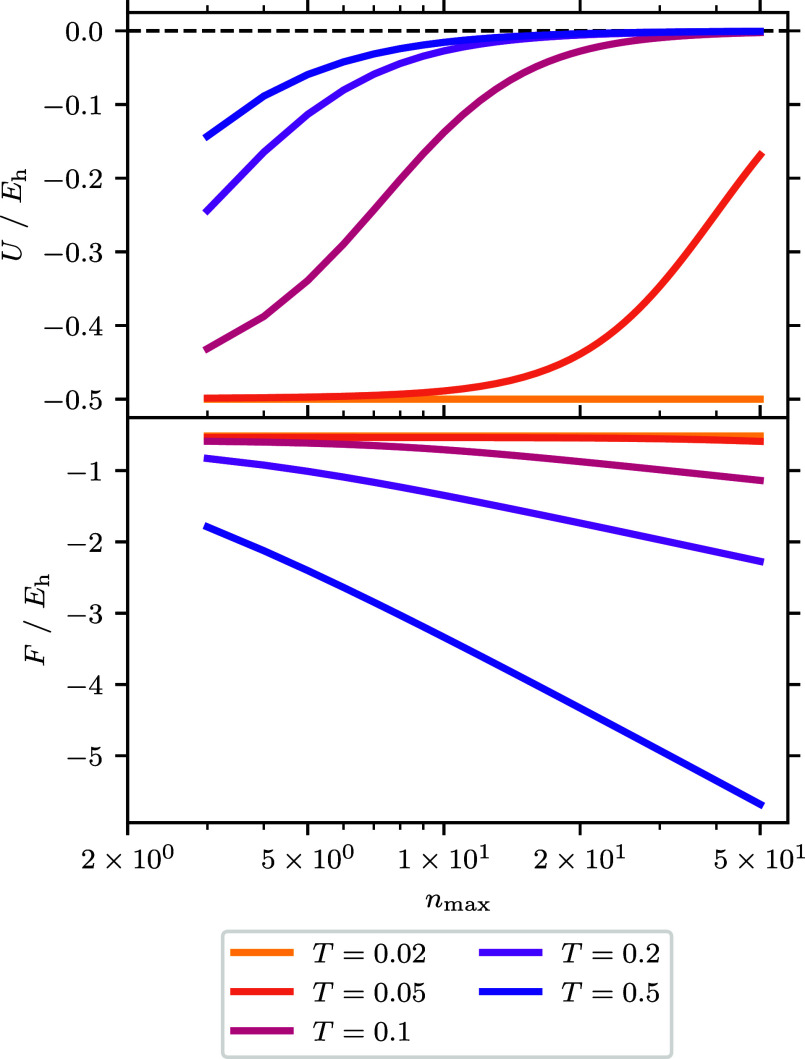
Internal energy and free energy of the hydrogen atom is shown as a function of the cardinal number cutoff, which is the equivalent of a finite basis set, for various temperatures.

Another problem involving basis set convergence at finite temperature is the free-energy divergence for atoms/molecules in a vacuum due to Rydberg states. This can be derived by starting from the analytical solution to the hydrogen atom Schrödinger equation. Using these, the internal energy for the hydrogen atom can be written
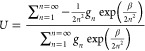
25The term for the degeneracy is: *g*_*n*_ = 2*n*^2^, including spin degeneracy. The result is that *U* = 0 for any finite temperature. In addition, the partition function alone diverges to infinity and the free energy goes to negative infinity.^a^ Replacing the *n* = ∞ top limit of the sum with a finite *n*_max_ yields a sum which is the equivalent to a finite basis set sum. A graph of the internal energy and free energy is shown in Figure 6 as a function of *n*_max_. This graph shows, numerically, that as the CBS limit is approached (*n*_max_ → ∞) the internal energy tends toward zero and the free energy tends to diverge to −∞.

We are interested in how the CBS convergence of an atom in a periodic box would behave compared to the hydrogen atom. The expectation for a hydrogen atom in a periodic box is that *U* → 0 would only be seen in the Ω → ∞ limit. Calculations were performed in PBC for two cubic volumes with *L* = 3 Å and *L* = 8 Å equivalent to Ω = 182.2 *a*_0_^3^ and Ω = 3455.2 *a*_0_^3^ respectively. Plane waves were used to compute Bloch orbitals, though it has been shown that approximate natural orbitals can greatly reduce the amount of BSIE in calculations at *T* = 0.^31^ We took caution to check the excited state spectrum looked reasonable at the HF level by counting the number of bound states. No effort was made to converge finite size effects and so that is likely a source of error in the Ω = 182.2 *a*_0_^3^ calculation. We anticipate the finite size effects will minimally impact our observations for larger *M* as the finite size error and BSIE coming from different length scales.

**Figure 7:**
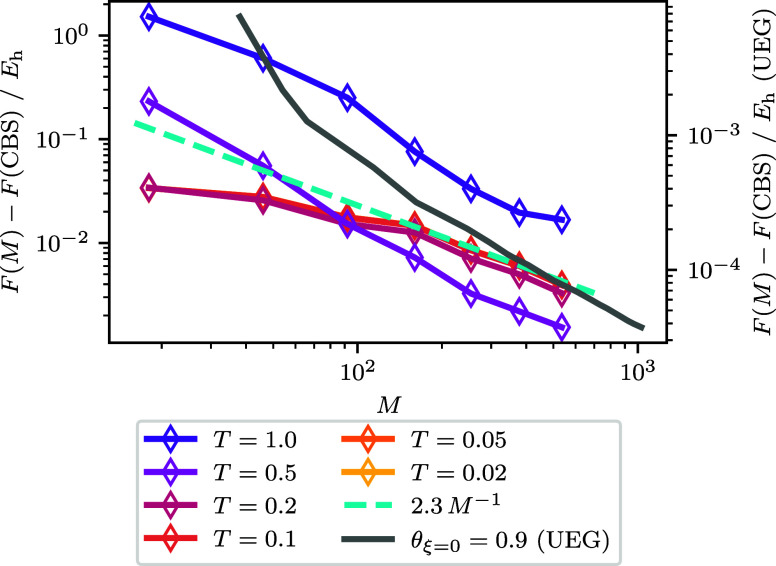
Free energy difference to the best CBS estimate for the helium atom under PBC (Ω = 182.2 *a*_0_^3^) as a function of spin–orbitals. The line corresponding to the UEG are for a density *r*_s_ = 1.

**Figure 8:**
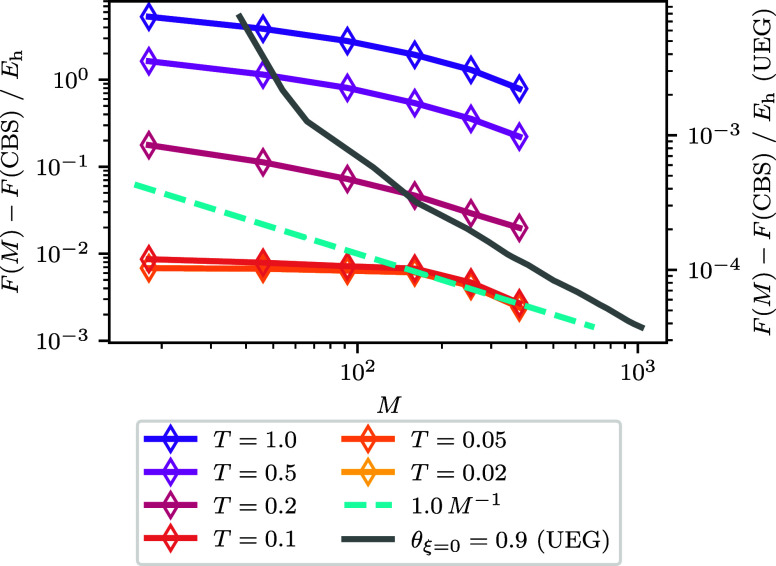
Free energy difference to the best CBS estimate for the helium atom under PBC (Ω = 3455.2 *a*_0_^3^) as a function of spin–orbitals. The line corresponding to the UEG are for a density *r*_s_ = 1.

Figures 7 and 8 shows the CBS convergence for the free energy for each volume at a variety of temperatures. Each line has a shape that eventually trends toward *M*^–1^ with enough basis functions. For Ω = 182.2 *a*_0_^3^, below *T* = 0.1 *E*_h_. The lines are overlapped and so cannot be resolved separately, but they each converge to the CBS limit as *M*^–1^. Then, at higher *T*, the curves change shape slightly but still trend toward the *M*^–1^ relationship in general. In the case of *T* = 1.0 we are able to see the cross-over between the more complex exponential relationship and *M*^–1^ at roughly *M* = 200. This evolution in the curves across temperature is similar to what we saw for the *r*_s_ = 1 UEG in Figure 5. For Ω = 3455.2 *a*_0_^3^, the trends is somewhat different. There is a very slow convergence to the point where *M*^–1^ is a good fit to the curve, which seems to originate in near-ground-state calculations. Other authors have found this to be related to orbital optimization and favor natural orbitals.^31,45^ These trends are somewhat different than the UEG, which does not require orbital optimization. This is consistent with observations by others that orbital optimization is important for converging BSIE efficiently.^31,45^

### 4.5 Basis Set Dependence for Distribution Functions

Distribution functions are important in terms of basis set convergence. They can be analyzed to better understand the CBS convergence and they can also be fitted to functions and interpolated to find CBS corrections. For the thermodynamic limit examples of the fit-and-interpolate approach can be found for finite temperature and ground-state calculations.^33,75−77^ However, they are also important because they are related to experimental observables^78,79^ and phase changes; the UEG structure factor has also been examined extensively in the study of warm dense matter.^80^

**Figure 9:**
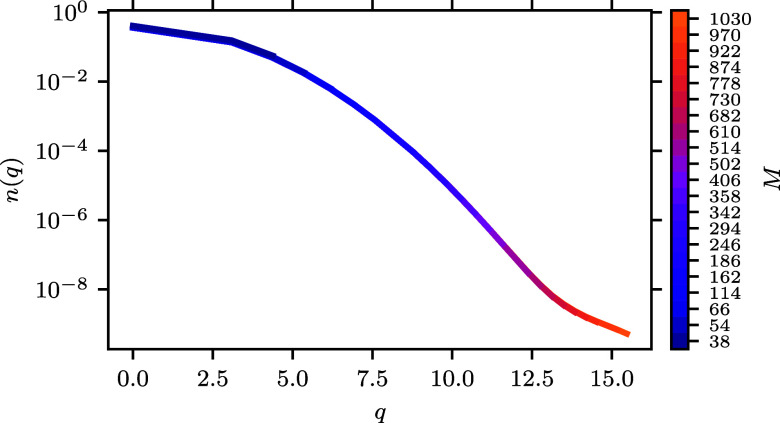
Momentum distribution as a function of momentum for the *r*_s_ = 1 UEG system at θ_ξ=0_ = 2.5.

Figure 9 shows the momentum distribution (*n*(*q*)) for a range of basis sets at θ_ξ=0_ = 2.5. We chose this temperature because, above, the kinetic energy showed exponential convergence in this regime so we expected that this would be the region where *n*(*q*) would vary significantly with varying basis set. These are plotted with a color map that shows the change in *n*(*q*) as more *M* is added. In the case of Figure 9, the larger basis set lines are added behind the smaller ones. Larger basis sets add new *q* values at larger *q* and this extends in the line with a new color. There is not a change in color over the rest of the line because *n*(*q*) does not generally change, at least by visual inspection.

**Figure 10:**
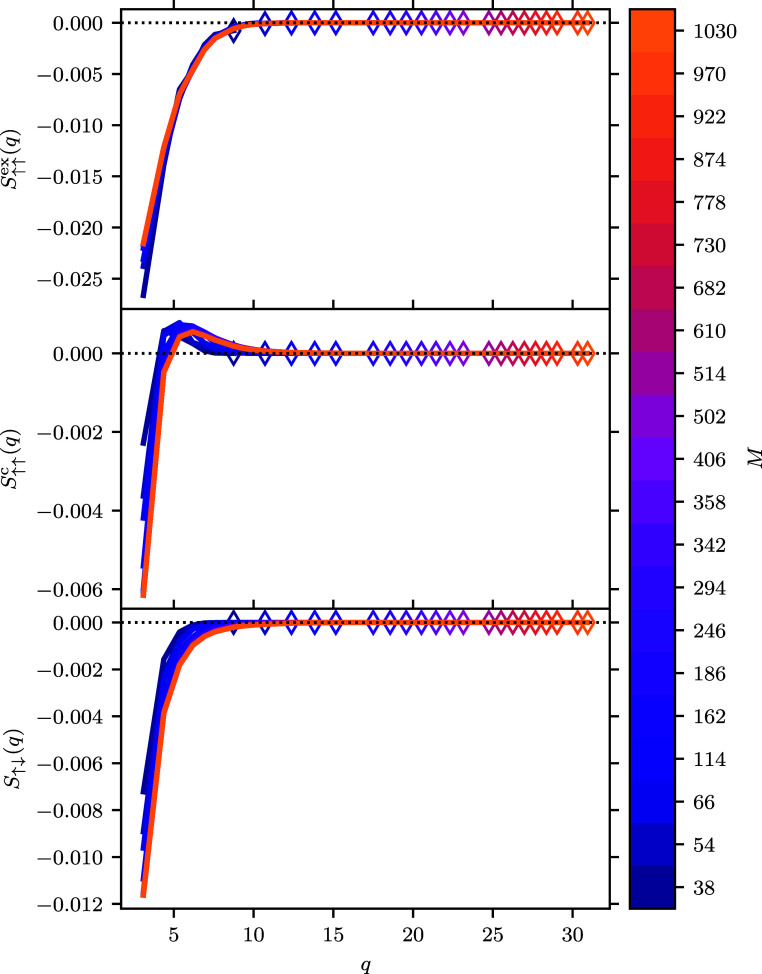
SSF components as a function of momentum transfer for the *r*_s_ = 1 UEG system at θ_ξ=0_ = 2.5. The marker indicates the maximum momentum transfer included in the structure factor due to the finite basis set.

Figure 10 shows the three components which contribute to the SSF for a range of basis sets at θ_ξ=0_ = 2.5. This temperature, chosen for *n*(*q*), is also appropriate for *S*(*q*) because there are significant contributions from all spin-polarizations at this temperature. These are plotted in such a way that the changing color map indicates the direction for convergence and markers are used to indicate where the largest *q* is present for a given *M*. In Figure 10, the larger basis sets are plotted on top of the smaller ones to highlight how different parts of the curve converge. As before, addition of more points in *q* as the basis set is increased in size is expected. On this graph, we can see that points along *S*(*q*) do change as basis functions are added. This is perhaps most significant in the case of *S*_↑↑_^c^(*q*), where addition of more basis functions changes the location and height of the peak where *S*_↑↑_^c^(*q*) > 0. That the different spin contributions converge at different rates is consistent with observations made from energy calculations by other authors.

## 5 Conclusions

We have investigated the basis set convergence behavior for thermodynamic quantities across a range of *T* using ft-FCI for a range of two-electron systems. In the UEG, we found extensive numerical evidence demonstrating the competition between *K* and *V* in *U*.^24^ Separating out *V* into its component parts, it was observed that *E*_↑↑_^ex^ mostly approaches the CBS limit from below, while *E*_↑↑_^c^ and *E*_↑↓_^c^ mostly approach from above. This observation of *E*_↑↑_^ex^ agreed with a deduction made by a previous study.^34^ At low *T*, *E*_↑↑_^c^ and *E*_↑↓_^c^ dominate convergence in *V*, while at higher *T*, *E*_↑↑_^ex^ competes. The momentum distribution and SSF for the UEG were also briefly explored, with their different components converging at different rates. We believe these quantities are exceptionally rich in detail, much of which we could not address here, and as such will devote a followup study into their behavior.

We formulated one version of the free energy change caused by electron correlation and identified that this and the remainder of the free energy would converge monotonically to the CBS limit due to the canonical ensemble variational principle. We observed that a change in *U* that would give rise to nonvariationality in *F* would be counteracted by −*TS*. The convergence rate of *F* with *M* was also analyzed for the UEG. Provided *K* achieves exponential convergence, the convergence rate for *F* with *M* in either system is found to agree well with the *U*(*T* = 0) convergence rates (*M*^–1^).^24,25,43,81−84^ This suggests that *S* has a similar or smaller rate of convergence to the CBS compared to *U*. We saw similar trends in a helium atom that was treated with PBC, which is one way to overcome the Rydberg states causing a divergence in the CBS limit. This bodes well for the use of plane wave based Bloch functions for correlated calculations.

The limitations of this work are that we are studying thermodynamics with two electrons—our systems are far away from the infinite particle (or thermodynamic) limit. In the case of the helium atom, this is likely more of an approximation in the smaller box where the interaction between helium atoms would have been affected by finite size effects. The study of basis set effects often uses two particle systems which are subsequently applied to many-particle systems. The success of this approach is attributed to the similarity in the shape of the two-electron coalescence points (the interelectron cusp) between different systems. In the present study this would only apply to the correlation components of the energy. In solids, we can extend this further by suggesting that the CBS limit and the thermodynamic limit commute i.e. can be taken in either order. Whether these arguments are valid for systems with finite temperature remains an open question. Nonetheless, studying the two electron system allowed us to explore some key ideas in the basis set convergence of finite-temperature energies.

## Data Availability

For the purposes of providing information about the calculations used, files containing the data and example calculation scripts will be deposited at https://github.com/shepherd-group/ftCBS-Data. The data that supports the findings of this study are contained within the article.

## Appendix

### A. Calculating the Momentum Distribution and SSF

We calculate the momentum distribution as

26

The spin components for the momentum distribution are calculated with the density matrix

27

28

where *N*_*n*_(*q*) is the number of momentum vectors **q** contributing with a nonzero amount to the momentum *q* = |**q**|, *m* is an occupied orbital in |*D*_*i*_⟩, and *q*_*m*_ is the momentum for orbital *m*. The momentum distribution is related to the kinetic energy

29

where the momentum distribution is implicitly dependent on β through ρ̂(β).

We calculate the SSF using equations developed in ref (34) with simplifications for a two-electron system. In particular, a constant factor of 2 cancels with the electron number *N* = 2, and the permutation term is dropped as it will always be positive unity. We also partition the SSF into several components following ref (85). Then we find the equations as

30

where we write the parallel spin SSF as

31

The parallel spin SSF components are calculated from the density matrix as

32

33

where *q*_*mn*_ is the momentum transfer between orbitals *m* and *n*, and ρ_*ij*_ are the components of the density matrix in the Slater determinant basis.

The antiparallel SSF is calculated from the density matrix as

34

In these equations *m* and *n* are the occupied orbitals in |*D*_*i*_⟩, which are excited from, while *a* and *b* are the occupied orbitals in |*D*_*j*_⟩, which are excited into. Furthermore it is assumed that *n* > *m*, *b* > *a*, {*m*,*n*} ∉ |*D*_*j*_⟩, and {*a*,*b*} ∉ |*D*_*i*_⟩. The SSF are then used to evaluate the spin-dependent exchange and correlation energies through the expressions

35

36

37

where *V*(*q*) = 4π/(*L*^3^*q*^2^) for *q* > 0 and zero otherwise. These three energies are related to the potential energy like

38

with all three being dependent on β because ρ̂(β) is used to calculate the SSF.

### B. Relationship between Canonical Ensemble Quantities for Different Polarizations

Starting from these two expressions that are present in the main manuscript as eqs 6 and 7

39

40

The impact of adding *m*_S_ sectors is to add terms to *Z*(β).

To combine two *m*_S_ sectors in *Z*(β)

41

and the internal energies combines using these partition functions

Other quantities can be derived in the same way, though the entropy is more complex. This implies that the individual *m*_S_ contributions can be found if the partition function or free energy is available.

## Footnotes

a This is demonstrated in a pedagogical derivation in ref (59).
